# Climate Change Policies in 16 West African Countries: A Systematic Review of Adaptation with a Focus on Agriculture, Food Security, and Nutrition

**DOI:** 10.3390/ijerph17238897

**Published:** 2020-11-30

**Authors:** Raissa Sorgho, Carlos A. Montenegro Quiñonez, Valérie R. Louis, Volker Winkler, Peter Dambach, Rainer Sauerborn, Olaf Horstick

**Affiliations:** 1 Heidelberg Research to Practice Group, Heidelberg Institute of Global Health (HIGH), Heidelberg University Hospital, Heidelberg University, Im Neuenheimer Feld 365, 69120 Heidelberg, Germany; raissa.sorgho@uni-heidelberg.de (R.S.); carlos.montenegro@uni-heidelberg.de (C.A.M.Q.); Valerie.Louis@uni-heidelberg.de (V.R.L.); volker.winkler@uni-heidelberg.de (V.W.); peter.dambach@uni-heidelberg.de (P.D.); 2 Working Group on Climate Change, Nutrition & Health, Heidelberg Institute of Global Health (HIGH), Heidelberg University Hospital, Heidelberg University, Im Neuenheimer Feld 324, 69120 Heidelberg, Germany; rainer.sauerborn@uni-heidelberg.de

**Keywords:** climate change, policy, adaptation, West Africa, agriculture, food security, nutrition

## Abstract

Climate change strongly impacts the agricultural sector in West Africa, threatening food security and nutrition, particularly for populations with the least adaptive capacity. Little is known about national climate change policies in the region. This systematic review identifies and analyses climate change policy documents in all 16 West African countries: (1) What are the existing climate change adaptation policies publicly available? (2) Which topics are addressed? (3) How are agriculture and food security framed and addressed? Following PRISMA guidelines, PubMed and Google scholar as key databases were searched with an extensive grey literature search. Keywords for searches were combinations of “Africa”, “Climate Change”, and “National Policy/Plan/Strategy/Guideline”. Fifteen countries have at least one national policy document on climate change in the frame of our study. Nineteen policy documents covered seven key sectors (energy, agriculture, water resources, health, forestry, infrastructure, and education), and eight thematic areas (community resilience, disaster risk management, institutional development, industry development, research and development, policy making, economic investment, and partnerships/collaboration). At the intersection of these sectors/areas, effects of changing climate on countries/populations were evaluated and described. Climate change adaptation strategies emerged including development of local risk/disaster plans, micro-financing and insurance schemes (public or private), green energy, and development of community groups/farmers organizations. No clear trend emerged when analysing the adaptation options, however, climate change adaptation in the agriculture sector was almost always included. Analysing agriculture, nutrition, and food security, seven agricultural challenges were identified: The small scale of West African farming, information gaps, missing infrastructure, poor financing, weak farmer/community organizations, a shifting agricultural calendar, and deteriorating environmental ecology. They reflect barriers to adaptation especially for small-scale subsistence farmers with increased climate change vulnerabilities. The study has shown that most West African countries have climate change policies. Nevertheless, key questions remain unanswered, and demand for further research, e.g., on evaluating the implementation in the respective countries, persists.

## 1. Introduction

Climate change (CC) is recognised as a threat to health, nutrition, and the livelihood of populations.

Governments across the world have collectively negotiated and worked on solutions to mitigate and adapt to CC, and numerous treaties/agreements have been signed, from the Kyoto protocol in 1997 to the Paris agreement in 2015 [1,2,3]. Low- and middle-income countries are disproportionately affected by these impacts [4,5] and limited ability for planning, mitigation, and adaptation [6]. Hence, this study systematically determines whether West African countries have drafted and planned for adaptation through national CC policies.

CC policies are especially important for West African countries because CC is a new and amplifying risk factor for malnutrition/food security, particularly in countries with subsistence agriculture [7,8,9]. Preserving food security is particularly relevant for low-income countries where hunger and related productivity loss represent an enormous human and economic loss [10,11].

Between the years 2000 and 2050, CC is projected to cause additional 25.2 million chronically malnourished children <5 years, compared to a world without CC [12]. 10.5 million additional stunting cases will occur in sub-Saharan Africa, adding to the projected 41.7 malnourished children without CC occurring. This mirrors projected decreases in per capita food availability as median crop yields are expected to decrease due to CC by 0–2% per decade. At the same time, demand for crops is expected to increase by 14% per decade until 2050 [13,14,15]. 

CC policies should guide climate adaptation plans while addressing agricultural/food security. Here we focus on the West African region as a particularly vulnerable region for the effects of CC. Up to this date, no systematic analysis of existing CC policies exists for the West African region. Systematic analysis with the methods of systematic reviews have the advantage to systematically collect and analyse all relevant publications and data, ensuring that the analysis and following recommendations are not driven by expert opinion only [16].

Under this angle, this article systematically reviews the existing CC policies of 16 West African countries: Benin (BN), Burkina Faso (BF), Cape Verde (CV), Ivory Coast (IC), Gambia (GB), Ghana (GH), Guinea (GNA), Guinea-Bissau (GNB), Liberia (LB), Mali (ML), Mauritania (MR), Niger (NG), Nigeria (NGA), Senegal (SN), Sierra Leone (SL), and Togo (TG). The questions addressed are: (1) What are the existing CC adaptation policies publicly available? (2) Addressing which topics? (3) How are agriculture, food security, and nutrition framed and addressed?

## 2. Materials and Methods 

Following the Preferred Reporting Items for Systematic Reviews and Meta-Analyses (PRISMA) [16], data were extracted by two authors until November 2020. The databases included were very broad, to reflect that CC policies may be reported or published in a wide variety of sources. The following databases were used: (1) PubMed, (2) Google Scholar, (3) Grey literature sources: United Nations databases, websites of FAO, UNDP, UNFCCC, (4) 48 West African countries’ ministry governments websites (Ministry of Agriculture, Ministry of Environment, and Ministry of Health), and the (5) LSE and Grantham Research Institutes’ database. The search strategy applied was the same on all databases, only on Google Scholar, the first 200 relevant hits were screened.

Search terms were combinations of “Africa” and/or country names, “Climate Change” and “National Policy/Plan/Strategy/Guideline” (Table 1).

We used adaptation as “The process of adjustment to actual or expected climate change and its effects. In human systems, adaptation seeks to moderate or avoid harm or exploit beneficial opportunities” [17]. This means that adaptation is planned for autonomous adjustments of individual groups or institutions in ecological-socio-economic systems in response to climatic stimuli, in order to reduce the society’s vulnerability [18,19,20]. Policy documents are defined as internet-accessible plans, programmes, strategies, and guideline documents issued by national governments.

Inclusion criteria were: (1) National policy documents (2) of the 16 West African countries, (3) from any time-period, (4) publicly accessible, (5) in English, French, or Portuguese. 

Publicly accessible documents are defined as document free of charge in digital or print format, accessible through internet search or by request through in-country experts at the step of identifying the full text of policies. The policy documents were often written in multiple languages (including English), contained abstracts or executive summaries in English, and were catalogued or indexed in English.

**Figure 1:**
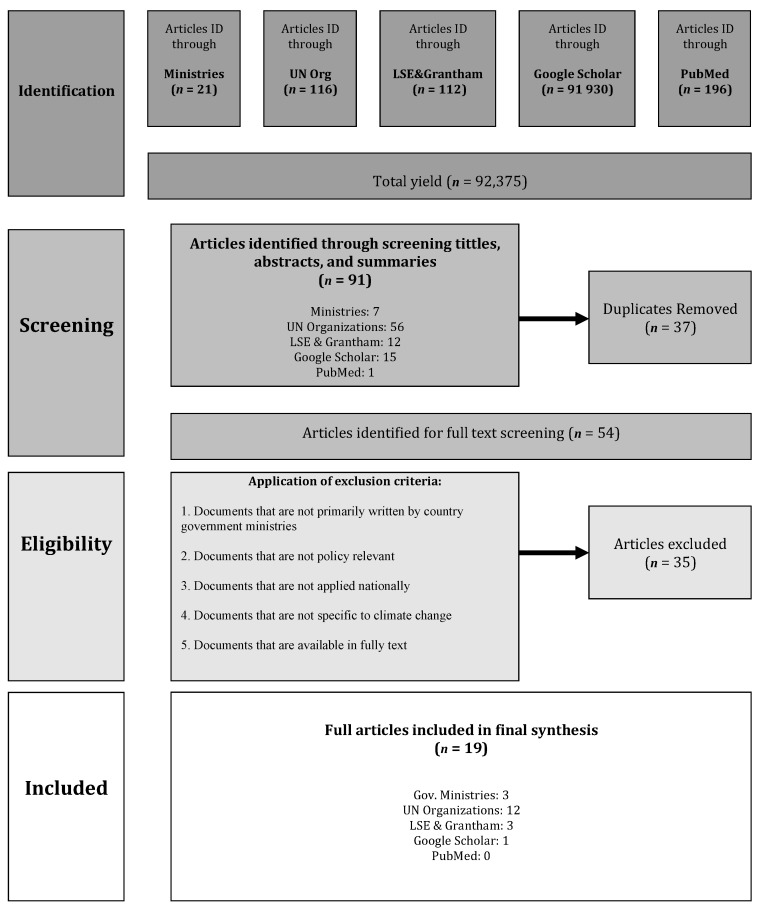
Flowchart of searches with the identification of policies, screening, eligibility, and inclusion.

Exclusion criteria were: (1) Not written by or in collaboration with country governments, (2) no policy relevance (international reports, UN communications, scientific publications), (3) not applied nationally, (4) not specific to CC, and (5) not available in full text (Figure 1). 

Data were extracted from the finally included policy documents into a pre-defined data extraction sheet, covering topics as general characteristics, aims, integration into other policies, characterisation of CC and mitigation, linking of CC and agriculture including adaptation strategies, linking of CC and nutrition/food security, including adaptation strategies (Supplementary Materials Table S1).

The extracted information was analysed by country, type of document, and subject of interest. The data were then synthesised based on key points emerging from the policy documents. The content of the data was then qualitatively analysed through overarching themes developed from the documents. Next, the technical guidelines for the national adaptation plan process of the UNFCCC was used as framework for policy comparison, and lastly, a quality assessment of the policies was conducted [21]. 

## 3. Results

This section covers (Section 3) descriptive analysis with (Section 3.1) Searches and included documents (Section 3.2) key sectors, (Section 3.3) thematic areas and a (Section 3.4) comparative analysis with (Section 3.4.1) agricultural adaptation challenges, (Section 3.4.2) nutrition and food Security challenges, (Section 3.4.3) agriculture, nutrition, and food adaption solutions, and (Section 3.5) policy document comparison. Policy identification codes (PID) identify each policy document.

### 3.1. Searches and Document Inclusion

Searches initially retrieved 92,375 hits at the identification phase. Through screening of titles, abstracts, and summaries, 91 relevant hits were retained from all databases, then 35 duplicates were removed. Of the 47 documents identified for full text screening, 37 were eliminated based on the exclusion criteria. Eight of the 37 excluded articles were due to the inaccessibility of the full text of policies (Figure 1, Table 2, Supplementary Materials Table S2). At the end of the search, the 19 final documents were derived from following sources: Twelve United Nations organization web bases (FAO, UNDP, UNFCCC), three from LSE and Grantham, three from government ministry websites, and one from Google Scholar (Figure 1, Table 2). 

The 19 included policy documents [22,23,24,25,26,27,28,29,30,31,32,33,34,35,36,37,38,39,40] originated from 14 countries. Guinea and Senegal had no relevant policy documents publicly available in the scope of this search, Benin, Ghana, Liberia, Mali, and Nigeria had more than one (Table 2). All included documents described the country, the listed agencies involved for document development, and specified aims/objectives. Seventeen of 19 documents mentioned other relevant national documents (laws, policies, reports, programmes) which were connected/relevant. Thirteen policies explained the effects of CC and elaborated on key scientific concepts as CC mitigation. Thirteen documents gave full explanations of CC mitigation, further explaining CC adaptation. Documents were often structured on key sectors and/or thematic areas. Seven common key sectors were identified across the policies: Energy, agriculture, water resources, health, forestry, infrastructure, and education (Table 3); and eight thematic areas emerged from the policy documents: Community resilience, disaster risk management, institutional development, industry development, research and development, policy making, economic investment, and partnerships/collaboration. Lastly, adaptation strategies were described for the above key sectors (Table 3).

### 3.2. Key Sectors

(I) Energy was discussed with a focus on electricity, petroleum, and renewable energies. More specifically, production, use, maintenance, and population need of energy and its different sources (PID: BN1, BN2, BF1, IC1, GB1, GH1, GH2, LB1, LB2, ML2, ML3, NG1, NGA1, NGA2, TG1).
(II) Agriculture was identified both as a key and vulnerable sector. Agriculture included animal husbandry, crops diversification, food granaries storage systems, and food security on a national level (PID: BN1, BN2, BF1, IC1, GB1, GH1, GH2, LB1, LB2, ML2, ML3, NG1, NGA1, NGA2, TG1).
(III) Water resource sections highlighted water use/management, desertification, fishing industry, and sometimes sanitation (PID: BN1, BN2, BF1, IC1, GB1, GH1, GH2, GNB1, LB1, LB2, ML1, ML2, ML3, ML4, MR1, NG1, NGA1, NGA2, TG1).
(IV) Health was one of the broader sectors, displaying a wide range of subtopics: Vector-borne and infectious diseases, malnutrition, heat, and human productivity, emphasising exacerbation of already existing differential social vulnerabilities (PID: BN1, BN2, BF1, IC1, GB1, GH1, GH2, GNB1, LB1, LB2, ML1, ML2, ML3, ML4, NG1, NGA1, NGA2, TG1).
(V) Forestry reiterated the importance of maintaining the environment, and the numerous ecological systems. Threats such as desertification and industrialisation were described in relation to sustainability and adaptation. (PID: BN2, BF1, IC1, GB1, GH1, GH2, GNB1, LB1, LB2, ML1, ML2, ML3, ML4, NG1, NGA1, NGA2, TG1).
(VI) Infrastructure sector centred on transport, housing, and safety. Focusing on deterioration of the above-mentioned systems, economic repercussions, and population wide effects if systems fail (PID: BN2, BF1, GB1, GH1, GH2, LB1, LB2, ML1, ML2, ML3, ML4, NGA1, NGA2, TG1).
(VII) Education mentioned three subtopics in relation to CC: (1) Education of relevant key local government members, (2) school-based education of children, (3) education of the general population (PID: BN1, BF1, GB1, GH1, GNB1, LB1, LB2, ML3, MR1, NGA1, NGA2, TG1).

### 3.3. Thematic Areas

Eight interconnected thematic areas were identified during the analysis of the policy documents. They were common amongst and throughout the documents, often utilised in combination with the seven key sectors (Table 3).

(i) Partnerships and collaboration were often used in preambles. Slogans and logos of partnering country ministries and organisations were mentioned. Most frequently, the United Nations Development Program (UNDP), the United Nations Framework Convention on Climate Change (UNFCCC), German International Cooperation (GIZ), the Embassy of Norway, and the Agency for Environment and Sustainable Development (AEDD) were mentioned. Continued partnership with these organisations was encouraged, emphasising building relations with surrounding countries, regional organisations, and regional groups. Public government agencies were encouraged to stimulate, motivate, and collaborate with private businesses/private sectors (PID: BN1, IC1, GB1, GH1, GH2, GNB1, LB1, LB2, ML1, ML2, ML3, ML4, MR1, NGA1, NGA2, TG1).
(ii) Industry development emerged with a focus on public and private sectors, involving these for growth/development. Private sector involvement in government and public initiatives such as generating renewable energy in-country was emphasised as a highly desirable outcome, as positive and sustainable development for industries. Industry development was described as a key contributor to the development of green economies, powered by growth in renewable energies and green technology (PID: BF1, IC1, GH1, LB2, ML1, ML3, ML4, MR1, NG1, NGA1, NGA2, TG1).
(iii) Research and development focused on advancement of green technology. Policies evoked research and development for innovation, both in science on CC, and technology to cope with it. The majority of documents summarised the current climate science conducted. Documents also outlined knowledge gaps, and areas requiring further exploration through research and studies, e.g., localised weather shifts, early warning systems development (PID: BN1, BN2, BF1, IC1, GB1, GH1, GNB1, LB1, LB2, ML1, ML2, ML3, ML4, MR1, NG1, NGA1, NGA2, TG1).
(iv) Institutional development focused on expanding the local/regional frameworks, supporting growth, for industry and research and on the local level. The rise of civil society groups centred on CC, and promotion of green employment, jobs, and careers. These opportunities should be created together with an equivalent workforce, meaning training/capacity building for promotion of climate conscious activities and lives. Educating and training members of the society strengthen the countries’ institutions and communities (PID: BN1, BN2, BF1, IC1, GB1, GH1, GH2, GNB1, LB1, LB2, ML1, ML2, ML3, ML4, MR1, NG1, NGA1, NGA2, TG1).
(v) Construction of resilient communities was a common subtopic across the documents, expressing the need for communities knowledgeable about CC effects. The first step was empowering communities to be agents of mitigation and to make use of adaptive strategies. Building resilient communities was also highlighted as essential for vulnerability reduction on a large scale: Resilient communities can stimulate more equitable social development (PID: BN1, BN2, GB1, GH1, LB1, LB2, ML1, ML3, ML4, MR1, NG1, NGA1, NGA2, TG1).
(vi) Disaster risk management is highlighted as directly related to managing, reducing, averting, and warning against the risks faced by communities/populations. Less resilient communities would be more affected by climate related disasters, increasing the vulnerability of already vulnerable populations. Drought, heat induced wildfires, and management of important coastlines are considered to be in need of allocating funds, for adequate preparedness and response strategies (PID: BN2, IC1, GB1, GH1, GH2, LB2, ML2, ML3, NGA1, NGA2, TG1).
(vii) Policy making revolved around promotion of all previously mentioned thematic areas on the political scene/at the policy making level. Fourteen documents state that political will makes a difference in motivating and advancing the creation of climate friendly policies (guidelines, programmes, plans, etc.). Political will and political promotion, along with governance, coordination, and integration, were also mentioned as essential at the implementation and financing stages of adopted CC policies (PID: BN1, BN2, BF1, IC1, GB1, GH1, GH2, LB1, LB2, ML1, ML2, ML3, ML4, MR1, NG1, NGA1, NGA2, TG1).
(viii) Economic investment was omnipresent. It was both an underlining factor in all key areas and cross-cutting throughout the thematic areas. This theme supports the allocation of adequate ministry budgets, which are described as increasing the possibilities of implementing proper CC adaptation measures. The implementation of adaptation measures would stimulate green industry development, nurturing green economy, and in turn fostering institutional development, partnership, and collaboration, stimulating further growth and economic revenue (PID: BN1, BN2, BF1, GB1, GH1, GH2, GNB1, LB1, LB2, ML1, ML2, ML3, ML4, MR1, NG1, NGA1, NGA2, TG1).

The key sectors and thematic areas in all policies were intertwined with discussions of the nation’s development policies and aims, most often framed in the context of socioeconomic/poverty-reduction development, rural development, and sustainable development. 

### 3.4. In Depth Analysis of Agriculture, Nutrition, and Food Security Adaptation

In addition to the key sectors/thematic areas, in depth examination of agriculture, nutrition, and food security adaptation within the general and sector-specific plans of the 19 policies yielded seven agricultural barriers. 

#### 3.4.1. Agricultural Adaptation Challenges

Agriculture was extensively discussed and interfaces with numerous thematic areas, with barriers and enabling factors. (1) Small scale farming, (2) information gaps, (3) lack of infrastructure, (4) insufficient finances, (5) weak organisation, (6) shifting agricultural calendar, and (7) ecological unsustainability were perceived as the most pressing barriers to agricultural sectors’ CC adaptability.

Small scale farming is one of the largest barriers. Small farms are disproportionately affected by CC, also limiting adaptation potential. The Mali policy document (PID: ML2) states: “… the sectors most vulnerable to climate change are, in order, agriculture, health, fishing, energy, water resources, livestock, forest-wildlife, habitat, transportation, industry and education. Smallholder farmers, along with artisans, are the most vulnerable populations…”

Farmers lack essential information for agriculture, such as weather/precipitation data and more advanced information, as new farming strategies. Farmers also lack up-to-date information on the shifting agricultural calendar, with enormous ramifications on the entire growing season. Ivory Coast’s policy document states (PID: IC1) “The mismatch between weather calendars and growing seasons poses a real problem for agricultural production…” Lacking financial resources are also a barrier to agricultural adaptation. Policies pointed to low financial resources as a challenge at household level and on all levels of government (PID: NG1, NGA1, ML4. GB1). Farmers have limited resources to invest in adaptation, and governments have limited budgets to support farmers and essential adaptation steps such as infrastructural work (PID: GH1, ML4, GB1). 

Farming communities are suffering from and contributing to deteriorating ecological structures of the land (PID ML2, NG1, GB1, GH1). Farming activities effect the lands’ ecology through pollution, depletion, impoverishment of soil, water, and forest, through unsustainable, intensive, and growing exploitation (PID GH1). Mali illustrates this (PID: ML2) “…in Mali there is a significant degradation of soils, vegetation and terrestrial ecosystems. Soil degradation results from natural phenomena (water and wind erosion) accentuated by destructive agricultural practices. These practices are linked to the strong demographic pressure that reaches 2.4% of annual growth, and to the lack of improvement in agricultural productivity…” 

Weak levels of farmer organisations were highlighted as a barrier, as their existence enables the sharing of useful data for updating. Without farmer associations/community groups, it is a challenge to foster grassroots community movements and enact larger-scale changes within communities (PID: BF1). 

#### 3.4.2. Nutrition and Food Security Challenges 

Food security (availability, access, utilization, and stability) is defined by the FAO as the situation in which “all people, at all times, have physical, social, and economic access to sufficient, safe, and nutritious food that meets their dietary needs and food preferences for an active and healthy life” [41,42,43]. Nourishment is a key factor, as food security is related to nutrition, and conversely, food insecurity to malnutrition (undernutrition and overnutrition) [44]. 

Nutrition and food security were seldom addressed as independent sectors, but as sub-sectors/affiliated sectors such as human health and social protection (PID: GB1), changing agriculture landscape (PID: BN2, BF1, ML1), policy programme pillars, or collaborative NGO/IGO programmes (PID: BN1, BN2). 

In Gambia’s (PID: GB1), a more holistic approach entailed how CC will impact components of food security-availability, accessibility, and utilisation. CC will worsen the current food insecurity situation as quoted in (PID: GB1): “Only 18% of Gambian households are considered to be food secure while the national malnutrition prevalence rate of 9.9% verges on emergency level.” This in combination with the “existing economic, environmental and health risks have translated into high levels of food and nutrition insecurity.” Additionally, in Ghana’s (PID: GH1), emphasis is on “Food security is a crucial issue. Ghana reduced hunger by nearly three-quarters between 1990 and 2004, but food security disparities affect the delivery of the country’s development objectives.” Although both documents (PID: GB1 and GH1) recognise the severity of food security, no action points were mentioned. 

Guinea Bissau included a list of targeted actions, essential for adapting to the changing nutritional landscape and guaranteeing food security: “(i) Setting up of a national security stock (ii) Setting up of cereal banks (silos) by peasants to guarantee a food reserve in all regions (iii) setting up of an Early Warning System against risks (iv) Strengthening of sensitization campaigns about the importance of diversifying eating habits” (PID: GNB1).

In Guinea Bissau’s (PID: GNB1), the food security concerns were not limited to rural households, but also urban/semi-urban settings: “The country has survived, at a painful cost for its populations, to cycles of chronic crises, characterized by a worsening of access to water for agricultural purposes, human and animal consumption; a marked fall in agricultural production, staple food items (rice in particular); a rise in costs of some foodstuff, particularly in urban and semi-urban centers, a deterioration in prices of cashew and cotton and an increase in food insecurity.” 

Several policy documents, like Nigeria’s (PID: NGA1), drew attention to inequalities “Climate change will significantly affect vulnerable groups because of a variety of factors, including low adaptive capacity, limited resources, and poverty.” Women and farmers are highlighted as especially vulnerable to CC, due to their dependence on natural resources for their livelihoods (PID: GH1 and LB1). 

#### 3.4.3. Agriculture, Nutrition, and Food Security Adaptation Solutions 

Although the agricultural sector faces challenges due to CC, the policies illustrate that there is also adaptation potential. This sector’s ability to adapt is extremely important, as agriculture is linked to both the food security and economic stability of nations, as illustrated in Burkina Faso (PID: BF1): “Burkina Faso’s economy is predominantly agricultural, …It is a subsistence agriculture based on food grains (sorghum, millet, maize) which alone occupy more than 88% of the planted area with average yields of around 850 kg/ha, dominated by small family farms of 3 to 6 ha on average.” Although the rates of farming are high, production from small plots remains low related to certain non-efficient farming methods (PID: ML2). Increasing the challenges is a lack of information (environmental, weather, adaptation) (PID: IC1). 

Some governments offer a two-pronged approach through reinforced promotion campaigns and the use of climate-smart agriculture (CSA).

The use of established methods under the umbrella of CSA can increase agricultural efficiency and production, as stated in Benin (PID: BN2) “The adoption of climate-smart agriculture (CSA), which relies on irrigated agriculture, the valorisation of local seeds and endemic breeds, the search for new, more adapted varieties, reliable climate information and a national policy of land tenure security” is potentially an adaption solution. 

Pairing CSA with population information/promotion are suggested in Mali (PID: ML1): (1) Strengthen access to information and sensitization of the rural population regarding the agro-climatic reference calendar for the planning of agricultural activities (time of ploughing and sowing, appropriate period of agricultural interventions, timing of the appearance of certain diseases…). (2) Promote the use of meteorological information by farmers to improve agricultural production (support, information sessions). (3) Promote and strengthen the use of seasonal forecast data in agriculture (support, information sessions).

Lack of information is coupled with lack of access to information. Upstream of the access problem, is a communication, knowledge sharing, and knowledge dissemination problem at all levels of government. Ghana plans to resolve this through training/capacity building at the local level, including the media (PID: GH1): “… the challenge of translating complex climate science into messages that will resonate with the wider public. Here, the media has a crucial role to play in conveying “why climate change matters to me.”” Although Ghana has taken steps for capacity-building at the district level, capacity-building within the local communities needs addressing. 

The knowledge/information gap is mirrored by a resource gap: Few farmers and communities can make improvements to their farming and their agricultural strategies with limited financial resources or limited governmental funds. Implementation of publicly and privately-run financial mechanisms like micro-financing schemes are solutions, as in Mali (PID: ML2): “Many actions mentioned to build a green and climate change resilient economy require the population to make investments, due to their low financial capacity. It is therefore necessary to promote the development of financial services, particularly microfinance…” These solutions, paired with saving and insurance services, could enable populations to manage CC risks. 

Lack or poor infrastructure poses further challenges: There is an infrastructural gap, in areas ranging from transport, like the construction of roads, to commerce, like cash crop storage facilities (PID: BN2, BF1). Infrastructural support can improve selling agricultural products throughout the year and improve access to local and international markets, and this is precisely why the Burkina Faso government is investing in the improvement of infrastructure in their adaptation efforts. Their policy states (PID: BF1): “The Government’s efforts relate to the commercial production sectors (cotton, fruit and vegetables, hides and skins, meat, etc.), the improvement of road infrastructures, etc., which are likely to favor the competitiveness of Burkinabé products in regional and international markets.” 

The definition of agricultural zoning is a further challenge: Some farming villages are expanding the boundaries of farmed lands with no regard to zoning rules and regulations as described in Guinea Bissau (PID: GNB1). Defining agricultural zones may minimise the multitude of problems arising from unplanned expansion, using toxic lands, plains prone to flooding, and unlawful use and occupation of pre-owned and private land. 

Farmer organisations are a further solution mentioned, for example, in Gambia’s (PID: GB1): “It is well recognized in Africa that secure land tenure and access rights are essential for enabling community-based adaptation, as well as harnessing any related mitigation co-benefits. The [policy] will initiate a process to identify and act upon key constraints to community-based adaptation, including land tenure and access rights.” 

The ecological effects/footprint of agriculture, especially in combination with livestock and animal husbandry, are outlined at the beginning of the CC policies and further elaborated on in the National Communication of each country. Enumerated solutions to reduce greenhouse gas (GHG) and adapt the agriculture methods include, for example, in Mali (PID: ML2): “Soil fertilization of soils should be organized mainly using agro-ecological solutions (improved fallow, composting, manure, organic fertilizers, bio-pesticides, agroforestry, cultural techniques such as minimum tillage or plant cover, etc.), which are generally less expensive, more easily accessible, and more effective in the long term.” 

### 3.5. Policy Document Comparison 

Using the Technical Guidelines for The National Adaptation Plan Process of the UNFCCC as a framework for comparison: (1) Laying the groundwork, (2) preparatory elements, (3) implementing strategy, and (4) reporting, monitoring, and review (Supplementary Materials Table S3), the following emerges [21]:

Over a third of the documents analysed (PID: BF1, GB1, LB2, GH1, GNB1, NGA1, NGA2, TG1) followed each of the four steps, covering everything from initiating the process in multilateral collaboration to taking full stock of data gaps, information and possible hurdles of both the policy document drafting process, and of the research in their respective countries. The policy documents also analysed the weakness in adaptation to CC, and the possibilities for addressing them, and integrating further adaptation methods. Under the implementation strategy, these policy documents fulfilled all requirements, seamlessly crossing from discussion on the prioritisation of mainstreaming and integration of adaptation at the national level to promotion and coordination at regional and local levels. The last points addressed in these policy documents were elaborating, reporting, monitoring, and evaluation plans. 

Policy documents from Ghana and Mali proved less developed in step 2—Preparatory elements and step 3—Implementation Strategy (PID: GH2, LB1, ML3, ML4) 

In step 3, most of the policy documents successfully prioritised climate change in the national planning and developed adaptation implementation strategies, but many did not push further in addressing the last two points. They were weak in demonstrating how their national adaptation plans have been or would be updated (PID: BN2, IC1, ML1 ML2, NG1). 

Furthermore, Benin (PID: BN1) specifies a strategic plan to develop and enforce knowledge of human resource personnel on the subject of green development and climate resilience. The strategy points to a missing framework and structure for growing the pool of qualified persons in the public sector, the private sector, and civil society. The document elaborates an implementation, monitoring, and evaluation plan, an institutional framework, and a 5-year action plan.

Mauritania (PID:MR1) has a strategic plan for environment and CC in their education system. In addition to discussing its environmental, geographic, and ecological situation, this strategy details the 3 levels of the education system. This provides the institutional framework for research and the overall goal of the policy document: “change in attitudes and behaviours of current and future generations vis-à-vis environmental issues and sustainable development” through integration and education at all school levels. 

Liberia (PID: LB1) has an action plan specifically focused on gender and climate change. The plan’s purpose is to “… ensure that gender equality is mainstreamed into Liberia’s climate change policies, programs and interventions” in order for both men and women to equally benefit from the nation’s CC initiatives.

## 4. Discussion

### 4.1. Comparing Structure and Quality of the Policy Documents

Comparing the quality of the policy documents, it is important to first note the difference in the types of documents analysed. In this publication, “policy document” was used as an umbrella term covering publicly available national plans, guidelines, programmes, and policies. Although different in their development processes, aims, and outcomes, these terms are often used inconsistently and interchangeably [45]. Only the inclusive search and analysis of all these types of policy documents, as we present here, give an appropriate overview of the current state of West African CC adaptation planning [46,47]. 

With the expected range of policy documents, the primary aim of comparison was (1) to use a defined process of development, including a comparison to the suggested four stages by UNFCCC, and (2) to adequately review and identify the CC vulnerabilities and adaptation opportunities in the country. 

(1) The majority of documents, regardless if “plan, guideline, program, or policy” followed a process similar to UNFCCC’s technical guidelines for the national adaptation plan: “(1) Laying the ground work, (2) Preparatory elements, (3) Implementing strategy and (4) Reporting, monitoring and review” [21]. Except for two, the policy documents started by defining the groundwork for various subjects to be addressed throughout the document. Next, the documents also established the nation’s current state and identified information and data gaps. The policy documents addressed preparatory elements by recounting the countries’ previous climate related efforts and highlighting difficulties ranging from barriers in the policy drafting process itself to difficulties in acquiring and utilizing essential research for CC related activities. The policy documents analysed adaptation strength and weaknesses for which developed strategies would be implemented. After touching on implementation, most of the documents engaged discussions on the prioritisation actions and mainstreaming and integration of adaptation. They proposed various plans and methods for the reporting, monitoring, and evaluation of adaptation measure of the prioritized actions. The policy documents were thorough in discussing and covering the key aspects of the four steps. The three documents with a different development approach also had independent goals. These documents were narrow in focus and aims, each addressing only one specific aspect/sector; human resources, education, and gender. Due to this focus, they bypassed the more general analysis aspects that all other policy documents address in steps 1 and 2. Although weak in their elaboration of the entire process, they were strong in identifying vulnerabilities and barriers, then outlining the opportunities for improvements. 

(2) The second quality assessment of the policy documents was performed through qualitative content analysis using structured extraction tables to review the adaptation vulnerabilities and opportunities in the policy documents. At a second stage of the content analysis, an additional focus was placed on agriculture, nutrition, and food security. The analysis illustrated that although the type of policy documents varied, the vast majority had similar content, simply under differing structures. All but two of the three divergent documents, (PID: BN1) and (PID:MR1), adequately elaborated on the country’s wholistic challenges and opportunities for adaptation. From this wholistic review, seven key sectors emerged as most relevant to changing climate. These seven key sectors also illustrate a consensus in the West African countries. These are the sectors commonly deemed most vulnerable to the effects of the changing climate. Therefore, they emerge as priorities for adaptation action, which could be best implemented through actions in the eight interconnected thematic areas.

A study published in 2015 *“An overview of nationally appropriate mitigation actions (NAMAs) and national adaptation programs of action (NAPAs) in Africa”* mirrors this finding [48]. Although the review had a focus on forestry, the publication’s assessment of adaptation priority areas of both East Africa and West Africa yielded a combination of the key sectors and thematic areas also highlighted in our systematic review. The Kojwang review enumerated eleven “adaptation priority areas”, which overlap all seven key sectors outlined in our review with the exception of the education and energy sectors. Although energy is not on the adaptation priority list, it is first on the reviews’ mitigation actions list and a focal point for NAPA projects. The education sector was not mentioned at all in the 2015 review. The eleven “adaptation priority areas” enumerated in the Kojwang review overlap some of the eight thematic areas identified in our review, except for economic investment, partnerships, and the development centred thematic areas. Research and development, institutional development, and industry development were not mentioned in the 2015 review. However, this is not surprising, as the NAMAs and NAPAs are more homogeneous than the array of policy documents our systematic review identified. This shows that outside the frame of NAPAs and NAMAs, West African countries have developed an array of policy relevant documents to address a growing variety of sectors and areas. Another difference between the two studies was the 2015 study finding that most projects within the NAPAs are for energy, early warning systems, disaster reduction, coastal protection, and food security. Food security was not found to take centre stage across the policy documents of our systematic review. 

### 4.2. Possibilities for Integrated Adaptation

The adaption actions, solutions, and plans in the 19 national CC policy documents of this systematic review can be analysed along the lines of previously defined eight thematic areas. The proposed actions under the Disaster and Risk Management theme were focused on agriculture strategies management and water systems management. The agricultural strategies emphasised both an adaptive strategies and risk hedging methods: Diversification of crops, selection of crops based on durability, selection of crop variety based on maturation time, introduction of modified seeds based on better growth patterns, lower water requirement, and disease resistance (PID: ML2). Water system adaptation plans in the policies ranged from the creation of man-powered irrigation systems, to the use of traditional planting and bordering methods such as Zai and Half-Moon technique for increased water retention, methods supported by literature on CC and adaptation [49]. Proposed actions for water management included creating micro-dams, small dykes, and water reserves adjacent to agricultural fields. Field-adjacent water reserves have a twofold purpose, (1) as a convenient source of water in the middle of agricultural fields, and (2) serving as a drainage system in case of flooding, limiting the inundation of planted fields [50,51]. All this would preferably be performed through a participatory, integrated watershed management approach, insuring thorough analysis of the interrelation between the use of multiple methods and their impact on other sectors (PID: GB1). 

The continuous development of agricultural and water management systems should be supported by industry, research, and development. Necessary research and development to better confront the changing climate and weather variables begins with the use of natural resources. The development of renewable energies, especially solar energy, not only for countries’ electric grid, dominated by city use, but also for powering of equipment in rural sectors [52,53]. Development and promotion of conservative farming methods could contribute to reversing the trend of spreading and sprawling of land use for farming [54]. Ecosystem rebuilding and conservation is a subcategory of research and development, incorporating adaptive methods such as agro-forestry [55,56]. This serves to integrate agriculture and forestry, benefiting adaptation and mitigation [57,58]. This thematic area also discusses research on climate resilient ecosystems, sustainability, and energy sources. Lastly, this thematic area emphasises the inclusion of concerned community members, such as farmer’s/women’s groups at the centre of research and development, as they are key to the adoption and implementation of all relevant results [59,60]. 

Literature shows that concerned community members should also be implicated directly in the adaptation planning and activities of governing bodies [61,62]. This is necessary for building community resilience. Community resilience activities branch from integrating CC in the education system, both for teachers and students, to creating community empowerment groups [63]. These policies support strengthening social support networks, groups, and associations, since they also serve as access points for providing the population with information and tools for adaptation and protection of individuals and communities [64]. The introduction of integrated farming, alternative livelihoods systems, and income diversification are also adaptation initiatives, increasing households’ and communities’ resilience [50,65]. 

Initiation, promotion, and subsistence of the above-mentioned activities come from a new base of workers, trained in climate sciences through institutional development. These would be agents trained for capacity building purposes, at all levels of country organisation. The documents acknowledged the creation and or expansion of extension services as favourable to bridge the gap between science and research, its understanding, and application locally [63]. A trained green workforce would be essential in relaying and disseminating accurate climate related information, while simultaneously engaging in trainings and workshops with community members, making the community its own agents of change [66]. 

In looking at the policies, at the higher levels of government, several adaptation related actions could be mandated, grouped mainly in two areas; first, expanding governmental services, and second, strengthening government systems. For this, national policy documents need to lead to action points and implementable initiatives; followed by the development of climate actions/projects at regional and local levels. An advisory board could be set up to monitor the development of adequate and relevant guidelines, regulations, and projects. Agro-advisory boards for example could support the production of reliable weather-based crop focused information (PID: GB1). Such a board could also advise on new measures, such as establishing local weather warning systems and scaling up early warning systems for events such as floods, droughts, or famines. Lastly, these agro-advisory boards could back the promotion of agro-business both in the public and private sector (PID: ML2). Partnerships, in the private and public sectors, backing and funding green adaptation programmes are in the strategic plan of most policies [63]. Other adaptation-focused plans include the development of small-scale banking and agro-banking, primarily for implementation at local levels (PID: BN2). This is linked to a concept of weather-based insurance schemes and programmes, run either by the governments or in collaboration with international organisations (PID: BN2, GB1, GH1, ML2). The creation of national food storage facilities is another possible economic activity, including food insurance schemes. 

### 4.3. Climate Change and Development

The relation between CC and development is both parallel and intertwined. This was evident in the policies discussion of socioeconomic/poverty-reduction development, rural development, and sustainable development. The effects of the changing climate have the potential to set the national development back in many of the West African countries, effectively undoing much of the progress across sectors from effecting meningitis epidemics in the health sector, destroying water systems in the infrastructure sectors, to pushing women, farming communities, and all other groups reliant on natural resources further into poverty [67,68,69]. As a result, discussions have been sparked on the interconnectivity of development and CC efforts. This intertwined connection becomes even more difficult when discussing the prioritization and funding of activities. 

Though some policy documents have stated that partial funding has already been secured for their intended adaptation activities, most required significant investment to launch and upscale adaptation plans and projects. The allocation of government budget lines in West African countries are proving insufficient, therefore much funding must come from the private sector, regional, international, and non-governmental organisations and other partners [70,71]. As global funding streams expand and shift to include the allocation of funds for climate oriented, climate sensitive, and specific activities and catastrophes, there is a shift away from some previously funded development-based activities [72]. Furthermore, there is growing confusion on the differentiation of these activities, pushing the need to find a way to determine which activities are climate resilience and management, which fall under development. One of the proposed methods for differentiating development and CC activities are the use of indicators [73,74]. We would argue that although the elaboration of development and CC (mitigation and adaptation) indicators can provide a platform to sort and distinguish the two, it is more important to identify areas in which integrated approaches can be utilized and capitalized [75]. Integration would increase efficiency of results and create more efficient use of currently limited resources. 

One of the next steps for West African countries will be to increase their access to adaptation funds. Such resources would allow them to address both urgent/immediate needs while continuing to monitor, evaluate, and improve current efforts and initiatives. The acquisition of funds would drive the West African region forward in its development and climate change adaptation agenda. 

### 4.4. Limitations of the Systematic Review 

The first limitation was the accessibility of CC policies. No comprehensive climate change policy data base exists for African countries. The London School of Economics and Political Science and The Grantham Research Institute on Climate Change and the Environment established a platform bridging the public to environment decrees, laws, and policies, but the database for African countries is not yet comprehensive, up-to-date, or linked to the policy texts. Searching for policy documents directly on national ministry websites proved equally limiting. Of the 48 Ministry web sites searched, only 34 were functional. The remaining 14 websites were dysfunctional in a variety of ways: From disconnected web addresses to websites indefinitely under construction. Of the functioning websites, few provided access to the policy text. 

As a result, most of the policies were identified through extensive grey literature source documents, in a two-step process of (1) identifying existing policies and (2) locating a copy of the full text. Had the full text of all policy documents been accessible, the final included policies would have increased by nine, totalling 24. The search for the missing nine full text documents was extended to include the networks of three experts who were also unable to locate the policies. 

The inaccessibility of policies constitutes a barrier for researchers, IGO, NGOs, and for citizens. This is an overarching difficulty in many sectors from health to economics to CC, in most low-middle income countries [76]. The public availability of policies can increase general citizen knowledge and understanding of government goals and priorities [77], encouraging public engagement, fostering transparency and stimulating accountability [78,79]. The availability and accessibility of such documents can also facilitate the monitoring of activities, not only within and between national ministries, but for all agencies interested in tracking CC adaptation progress of a country [80,81]. 

Lastly, this review excluded National Communications (NC), National Adaptation Plans (NAP), NAPA and NAMA for which LDCs were provided UNFCCC funding and training. Although this systematic review focused on documents drafted outside this frame, it is important to acknowledge their use as source documents in this review. All the selected West African countries have as of 2019 drafted and submitted an Initial NC and a First NC. Fourteen have submitted a Second NC, twelve a Third NC, and two have a Fourth NC. Thirteen of the sixteen countries have a NAP, four have a NAPA, four have a NAMA. A 2015 review by [48] already provides an overview of the NAMAs and NAPAs in Africa, therefore they were not re-analysed in this review. Instead, the NAP drafting guidelines were used as a framework for comparison, and the 2015 review provided for adaptation action and priority comparison. The policies in this systematic review proved more wide ranging in the scope of subjects and more diverse in the proposed areas of adaptation options, as they were not limited by a prescribed standardized format, framework, and process. 

## 5. Conclusions

In conclusion, the systematic literature review aimed to answer the following three questions:

(1) What are the existing CC adaptation policies publicly available?

The systematic review of CC policies in West Africa identified 19 policies in 12 countries. Guinea had no policies in the frame of this review, Cape Verde, Sierra Leone, and Senegal had at least one policy in the frame of the review, but the full text was not publicly available and could not be located.

(2) Addressing which topics?

The policy documents effectively described the national situation, the agencies involved in drafting the document, and the aims/vision of the policies. The documents (17 of the 19) also strived to link and integrate other relevant national reports, laws, programmes, and policies. This is one of the initial steps in policy integration, which 13 policy documents discussed or stated as a goal. 

The policy documents converged on seven key sectors: Energy, agriculture, water resources, health, forestry, infrastructure, and education. The policy documents discussed these key sectors in the context of eight thematic areas: Community resilience, disaster risk management, institutional development, industry development, research and development, policy making, economic investment, and partnerships/collaboration.

(3) How are agriculture, food security, and nutrition framed and addressed?

Nutrition and food security challenges and adaptation were not highlighted as key points. They were seldom mentioned as an independent goal and most often under the umbrella of other sectors or areas, such as health, agriculture, and climate resilient systems.

Agriculture was a focal point of the policy documents. It was highlighted as a top three GHG emitting source in West African countries. The agricultural sector was identified as a key sector and as one of the most vulnerable sectors to CC, but it was also highlighted as a sector with immense possibilities and potential for adaptation. 

The primary challenges of CC agricultural adaptation were: The small scale of farming, gap of information, lack of infrastructure, insufficient finances, weak organisation, a shifting agricultural calendar, and ecological unsustainability. The enumerated solutions ranged greatly, from solidification of farmer groups, and training of climate community agents, to the use of modified seeds, weather-based insurances, and agro-forestry. 

There was also a plethora of proposed adaptation solutions for the other six key sectors, ranging from participatory integrated watershed management approaches, engagement of public–private partnerships in micro-financing and insurance schemes, construction of food-bank infrastructures, integration of CC in school curricula, training of a green human resource workforce, to serve as agents of change in communities, and also to fill a multitude of new jobs in growing renewable and green energy economies. 

In addition to addressing the research questions, this systematic review compared national climate change policies using the PRISMA and UNFCCC framework, determining where on the Climate Change policy development spectrum 16 West African countries lie. This manuscript is a first in the expanding realm and intersection of climate change, policy, and health, creating a scientific bridge (between well-established methods and the newly emerging subject of climate adaptation) into new bodies of knowledge.

The results of the review can inform the policy process in the West African countries. It clearly underlines the variability of the policies, and this is probably reflected in any implementation process.

Lastly, the added value of the review is the more scientific approach for document analysis, searching and analysing in a prescribed way with the methodology of systematic reviews, making the analysis and its key conclusions stronger. 

Next steps for research would be analysing the implementation of the identified CC policies. First, examining whether adaptation plans elaborated in these policies, were budgeted, developed, and implemented. Second, analysing the effect on agricultural, nutritional, and food security of the countries and their citizens.

## Figures and Tables

**Table 1:** Search phrase details for the systematic review.

Literature	Source	Search Phrases
Peer Reviewed	Google Scholar https://scholar.google.com	“Africa OR African” “Climate Change” “National Policy OR Policies” “Africa OR African” “Climate Change” “National Plan OR Plans” “Africa OR African” “Climate Change” “National Strategy OR Strategies” “Africa OR African” “Climate Change” “National Guideline OR Guidelines” “Country Name” “Climate Change” “National Policy OR Policies” “Country Name” “Climate Change” “National Plan OR Plans” “Country Name” “Climate Change” “National Strategy OR Strategies” “Country Name” “Climate Change” “National Guideline OR Guidelines”
	PubMed https://www.ncbi.nlm.nih.gov/pubmed/	(((Africa or African)) AND Climate Change) AND National Polic (((Africa or African)) AND Climate Change) AND National Plan (((Africa or African)) AND Climate Change) AND National Strateg (((Africa or African)) AND Climate Change) AND National Guideline (((Country Name)) AND Climate Change) AND National Polic (((Country Name)) AND Climate Change) AND National Plan (((Country Name)) AND Climate Change) AND National Strateg (((Country Name)) AND Climate Change) AND National Guideline
Grey	**Government Website** Ministry of Environment Ministry of Agriculture Ministry of Health Found using Google	**Ministry Webpage Information Tabs of each country:** Search each individual tab for relevant policies **Webpage Search Bar:** “National Policy” “National Plan” “National Strategy” “National Guidelines”
	**Research Institute** Grantham Research Institute on Climate Change and the Environment http://www.lse.ac.uk/GranthamInstitute/countries/	**Country Webpage Information Tabs:** Search each individual tab for relevant policies **Webpage Search Bar:** “Africa” “Climate Change” “National Policy” “Africa” “Climate Change” “National Plan” “Africa” “Climate Change” “National Strategie” “Africa” “Climate Change” “National Guidlines” “Country Name” “Climate Change” “National Policy” “Country Name” “Climate Change” “National Plan” “Country Name” “Climate Change” “National Stategie” “Country Name” “Climate Change” “National Guidlines”
	**United Nation Organizations** UNFCCC UNDP FAO https://unfccc.int http://www.adaptation-undp.org http://www.fao.org/faolex/country-profiles/en/	**UNFCCC–Main webpage Information Tabs:** Search each individual tab for relevant policies Read thought INDC, NC **UNDP–Climate Change Adaptation Portal:** Seach each individual country Read throught NAP, NAPA, NAMA **FAO–FOALEX Database** Search each individual country profile Read through policies, legislation, and international agreements

**Table 2:** Nineteen included policies, coded by country.

	Policy Identification (PID)	Policy Document Name
1	BN1	National Strategy for Strengthening Human Resources, Learning and Skills Development to Support Green, Low Emissions and Climate Resilient Development.
2	BN2	Low Carbon and Climate Change Resilient Development Strategy 2016–2025
3	BF1	The National Strategy for implementing the Climate Change Convention
4	IC1	National Climate Change Program
5	GB1	National Climate Change Policy of The Gambia
6	GH1	Ghana National Climate Change Policy
7	GH2	Guidebook for Mainstreaming of Climate Change and Disaster Risk Reduction for MMDAs
8	GNB1	National Programme of Action of Adaptation to Climate Changes
9	LB1	Climate Change Gender Action Plan for the government of Liberia
10	LB2	National Policy and Response Strategy on Climate Change
11	ML1	National Strategy on Climate Change
12	ML2	Strategic Framework for a Green Economy Resilient to Climate Change
13	ML3	National Policy on Climate Change
14	ML4	National Climate Change Strategy: National Climate Action Plan
15	MR1	Strategy for integrating the environment and climate change in the Mauritanian education system
16	NG1	National Policy on Climate Change
17	NGA1	National Adaptation Strategy and Plan of Action of Climate Change for Nigeria
18	NGA2	National Policy on Climate Change
19	TG1	National Plan of Adaptations to Climate Change of Togo

**Table 3:** Content of the analysed policy documents in seven key sectors and eight thematic areas.

**Key Sectors**		**Policy ID**																		
		**BN1**	**BN2**	**BF1**	**IC1**	**GB1**	**GH1**	**GH2**	**GNB1**	**LB1**	**LB2**	**ML1**	**ML2**	**ML3**	**ML4**	**MR1**	**NG1**	**NGA1**	**NGA2**	**TG1**
1	Energy	*	*	*	*	*	*	*	NMD	*	*	NMD	*	*	NMD	NMD	*	*	*	*
2	Agriculture	*	*	*	*	*	*	*	*	*	*	*	*	*	*	D	*	*	*	*
3	Water Resources	*	*	*	*	*	*	*	*	*	*	*	*	*	*	*	*	*	*	*
4	Health	*	*	*	*	*	*	*	*	*	*	*	*	*	*	D	*	*	*	*
5	Forestry	D	*	*	*	*	*	*	*	*	*	*	*	*	*	M	*	*	*	*
6	Infrastructure	NMD	*	*	M	*	*	*	D	D	*	*	*	*	*	M	M	*	D	*
7	Education	*	NMD	*	M	*	*	M	D	D	D	NMD	NMD	*	NMD	*	D	*	M	M
**Thematic Areas**		**Policy ID**																		
		**BN1**	**BN2**	**BF1**	**IC1**	**GB1**	**GH1**	**GH2**	**GNB1**	**LB1**	**LB2**	**ML1**	**ML2**	**ML3**	**ML4**	**MR1**	**NG1**	**NGA1**	**NGA2**	**TG1**
1	Community Resilience	*	*	M	NMD	*	*	D	D	M	D	*	NMD	*	*	*	*	*	*	M
2	Disaster risk Management	M	*	NMD	*	*	*	*	NMD	NMD	D	M	*	*	M	M	M	*	*	M
3	Institutional Development	*	*	*	*	*	*	*	*	M	*	*	*	*	*	*	*	*	*	M
4	Industry Development	M	M	D	*	M	*	M	NMD	NMD	*	*	M	*	*	*	*	*	*	D
5	Research & Development	*	*	*	*	*	*	NMD	D	M	*	*	M	*	*	*	*	*	*	M
6	Policy Making	*	*	*	*	*	*	*	NMD	M	*	*	*	*	*	*	*	*	D	D
7	Economic Investment	*	*	*	NMD	*	*	*	D	M	*	*	*	*	*	*	*	*	*	M
8	Partnerships/ Collaboration	*	M	M	*	*	*	*	*	M	*	*	*	*	*	*	M	*	*	M

* (asterisk): The sector/area is highlighted as central and or key in the policy document; **D** (Discussed): The sector/area is discussed often in different ways and parts of the policy document; **M** (Mentioned): The sector/area is mentioned or briefly touched upon in the policy document; **NMD** (Not mentioned-discussed): The sector/area is not mentioned or discussed in the policy document.

## Supplementary Materials

The following are available online at https://www.mdpi.com/1660-4601/17/23/8897/s1, Table S1: Extract of evidence table. Table S2: List of relevant policies whose full text could not be identified. Table S3: Summary of the steps for the technical guidelines for the NAP process.

## References

[1] UNFCCC COP21. The Paris Agreement to the United Nations Framework Convention on Climate Change (COP21). https://unfccc.int/process-and-meetings/conferences/past-conferences/paris-climate-change-conference-november-2015/cop-21.

[2] UNFCCC. COP8 The Delhi Declaration: Eighth session of the Conference of the Parties (COP8). https://unfccc.int/process-and-meetings/conferences/past-conferences/new-delhi-climate-change-conference-october-2002/cop-8.

[3] UNFCCC. COP3 Kyoto Protocol to the United Nations Framework Convention on Climate Change (COP3). http://unfccc.int/essential_background/kyoto_protocol/items/2830.php.

[4] Woodward A., Smith K. (2014). Chapter 11. Human Health: Impacts, Adaptation, and Co-Benefits. Climate Change 2014: Impacts, Adaptation, and Vulnerability. Part A: Global and Sectoral Aspects. Contribution of Working Group II to the Fifth Assessment Report of the Intergovernmental Panel on Climate Change.

[5] IMF Seeking Sustainable Growth: Short-Term Recovery, Long-Term Challenges. https://www.imf.org/en/Publications/WEO/Issues/2019/08/31/World-Economic-Outlook-October-2017-Seeking-Sustainable-Growth-Short-Term-Recovery-Long-Term-45123.

[6] Smith J.B., Klein R.J.T., Huq S. (2003). Climate Change, Adaptive Capacity and Development.

[7] Phalkey R., Aranda-Jan C., Marx S., Höfle B., Sauerborn R. (2015). Systematic review of current efforts to quantify the impacts of climate change on undernutrition. Proc. Natl. Acad. Sci. USA.

[8] Nelson G., Rosegrant M.W., Koo J., Robertson R., Sulser T., Zhu T., Ringler C., Msangi S., Palazzo A., Batka M. (2009). Climate Change: Impact on Agriculture and Costs of Adaptation.

[9] Lobell D.B., Burke M.B., Tebaldi C., Mastrandrea M.D., Falcon W.P., Naylor R.L. (2008). Prioritizing Climate Change Adaptation Needs for Food Security in 2030. Science.

[10] Blössner M., de Onis M., World Health Organization (2005). Malnutrition: Quantifying the Health Impact at National and Local Levels.

[11] African Union (2015). The Cost of Hunger in Africa: The Social and Economic Impact of Child Undernutrition in Burkina Faso. The Cost of Hunger in Africa.

[12] Nelson G.C., Rosegrant M.W., Palazzo A., Gray I., Ingersoll C., Robertson R., Tokgoz S., Zhu T., Sulser T.B., Ringler Msangi S. (2010). Food Security, Farming and Climate Change to 2050: Scenarios, Results, Policy Options.

[13] Challinor A.J., Porter J.R., Xie L., Cochrane K., Howden S.M., Iqbal M.M., Lobell D.B., Travasso M.I., Pramod Aggarwal K.H. (2014). Food security and food production systems. Climate Change 2014: Impacts, Adaptation, and Vulnerability. Part A: Global and Sectoral Aspects.

[14] Whitmee S., Haines A., Beyrer C., Boltz F., Capon A.G., de Souza Dias B.F., Ezeh A., Frumkin H., Gong P., Head P. (2015). Safeguarding human health in the Anthropocene epoch: Report of The Rockefeller Foundation-Lancet Commission on planetary health. Lancet.

[15] Lloyd S.J., Kovats R.S., Chalabi Z. (2011). Climate change, crop yields, and undernutrition: Development of a model to quantify the impact of climate scenarios on child undernutrition. Environ. Health Perspect..

[16] Moher D., Liberati A., Tetzlaff J., Altman D.G., Prisma Group (2009). Preferred Reporting Items for Systematic Reviews and Meta-Analyses: The PRISMA Statement. PLOS Med..

[17] IPCC (2014). The Fifth Assessment Report of the IPCC. Assessment Report.

[18] Pielke R. (1998). Rethinking the role of adaptation in climate policy. Global Environ. Chang..

[19] Fankhauser S., Smith J.B., Tol R. (1999). Weathering climate change: Some simple rules to guide adaptation decisions. Ecol. Econ..

[20] Smit B., Wandel J. (2006). Adaptation, adaptive capacity and vulnerability. Glob. Environ. Chang..

[21] UNFCCC NAP (2012). National Adaptation Plans: Technical Guidelines for the National Adaptation Plan Process.

[22] (2013). BN1. Stratégie Nationale de Renforcement des Ressources Humaines, de L’apprentissage et du Développement des Compétences Pour Favoriser un Développement Vert, Faible en Émissions et Résilient aux Changements Climatiques.

[23] BN2 (2016). Stratégie de Développement à Faible Intensité de Carbone et Résilient aux Changements Climatiques 2016–2025.

[24] BF1, Secrétariat Permanent du Conseil National pour la Gestion de l’Environnement (2001). Stratégie Nationale de Mise en Oeuvre de la Convention sur les Changements Climatiques.

[25] IC1, Direction Générale de l’environnement (2014). Programme National Changement Climatique.

[26] GB1 National Climate Change Policy of the Gambia. https://www.google.com.hk/url?sa=t&rct=j&q=&esrc=s&source=web&cd=&cad=rja&uact=8&ved=2ahUKEwiV4K6FlantAhXCaN4KHfbRCZAQFjACegQIAxAC&url=http%3A%2F%2Fwww.lse.ac.uk%2FGranthamInstitute%2Fwp-content%2Fuploads%2Flaws%2F8109.pdf&usg=AOvVaw2tLEUby_J6bz5NQnyMmIoX.

[27] GH1, National Climate Change Committee (2013). Ghana National Climate Change Policy.

[28] GH2 (2010). Integrating Climate Change and Disaster Risk Reduction into National Developement Policies and Planning in Ghana.

[29] GNB1 (2006). National Programme of Action of Adaptation to Climate Changes.

[30] LB1 (2012). Climate Change and Gender Action Plan for the Government of Liberia.

[31] Liberia E.P.A.o., LB2 (2018). National Policy and Response Strategy on Climate Change.

[32] ML1, Secretariat General (2011). Stratégie Nationale Changements Climatiques.

[33] ML2 (2011). Cadre Stratégique Pour une Economie Verte et Résiliente aux Changements Climatiques.

[34] ML3, Agence de l’Environnement et du Développement Durable (2011). Politique Nationale sur les Changements Climatiques.

[35] ML4, Agence de l’Environnement et du Développement Durable (2011). Stratégie Nationale Changements Climatiques: Plan d`Action National Climat.

[36] MR1, Ministère Delegue Aupres Du Premier Ministre Charge De L’environnement et Du developpement Durable (2012). Stratégie D’intégration de L’environnement et des Changements Climatiques Dans le Système Éducatif Mauritanien.

[37] NG1, Le Conseil National pour l’Environnement et le Développement durable (2012). Politique Nationale en Matière de Changements Climatiques.

[38] NGA1, Building Nigeria’s Response to Climate Change (2001). This National Adaptation Strategy and Plan of Action on Climate Change for Nigeria.

[39] NGA2, Ministry of Environment (2013). National Policy of Climate Change.

[40] TG1 (2016). Plan National d’Adaption aux Changements Climatiques du Togo.

[41] FAO (2001). The State of Food Insecurity in the World 2001. Food Insecurity: When People Live with Hunger and Fear Starvation.

[42] FAO (2018). The Future of Food and Agriculture: Alternative Pathways to 2050.

[43] FAO (2018). Combining Agricultural Biodiversity, Resilient Ecosystems, Traditional Farming Practices and Cultural Identity.

[44] Cheikh Mbow C.R., Noureddine Benkeblia A.C., Khan A., Porter J. (2019). Chapter5: Food Security. Final Government Distribution.

[45] WHO (2010). A Framework For National Health Policies, Straregies and Plans.

[46] Fischer F., Miller G.J. (2007). Handbook of Public Policy Analysis: Theory, Politics, and Methods.

[47] Pencheon D., Guest C., Melzer D., Gray J.M., Korkodilos M., Wright J., Tiplady P., Gelletlie R. (2006). Oxford Handbook Of Public Health Practice.

[48] Kojwang H.O., Larwanou M. (2015). An overview of nationally appropriate mitigation actions (NAMAs) and national adaptation programmes of action (NAPAs) in Africa. Int. For. Rev..

[49] Nouhoun Z., Dossa L., Schlecht E. (2014). Climate change and variability: Perception and adaptation strategies of pastoralists and agro-pastoralists across different zones of Burkina Faso. Reg. Environ. Chang..

[50] Below T., Artner A., Siebert R., Sieber S. (2010). Micro-Level Practices to Adapt to Climate Change for African Small-Scale Farmers A Review of Selected Literature.

[51] Mwungu C.M., Kizito F., Mwongera C., Koech N., Odhiambo C. (2019). Household Survey Data of Integrated Land and Water Management for Adaptation to Climate Variability and Change in West Africa.

[52] Mohammed Y.S., Mustafa M.W., Bashir N. (2013). Status of renewable energy consumption and developmental challenges in Sub-Sahara Africa. Renew. Sustain. Energy Rev..

[53] Ouedraogo N.S. (2017). Africa energy future: Alternative scenarios and their implications for sustainable development strategies. Energy Policy.

[54] Kimaru-Muchai S.W., Ngetich F.K., Baaru M., Mucheru-Muna M.W. (2020). Adoption and utilisation of Zai pits for improved farm productivity in drier upper Eastern Kenya. J. Agric. Rural Dev. Trop. Subtrop. (JARTS).

[55] Lasco R.D., Delfino R.J., Catacutan D.C., Simelton E.S., Wilson D.M. (2014). Climate risk adaptation by smallholder farmers: The roles of trees and agroforestry. Curr. Opin. Environ. Sustain..

[56] Mbow C., Van Noordwijk M., Luedeling E., Neufeldt H., Minang P.A., Kowero G. (2014). Agroforestry solutions to address food security and climate change challenges in Africa. Curr. Opin. Environ. Sustain..

[57] Mbow C., Smith P., Skole D., Duguma L., Bustamante M. (2014). Achieving mitigation and adaptation to climate change through sustainable agroforestry practices in Africa. Curr. Opin. Environ. Sustain..

[58] Verchot L., Van Noordwijk M., Kandji S., Tomich T., Ong C., Albrecht A., Mackensen J., Bantilan C., Anupama K.V., Palm C. (2007). Climate change: Linking adaptation and mitigation through agroforestry. Mitig. Adapt. Strateg. Glob. Chang..

[59] Idrissou Y., Assani A.S., Baco M.N., Yabi A.J., Traoré I.A. (2020). Adaptation strategies of cattle farmers in the dry and sub-humid tropical zones of Benin in the context of climate change. Heliyon.

[60] Alhassan S.I., Kuwornu J.K., Osei-Asare Y.B. (2019). Gender dimension of vulnerability to climate change and variability. Int. J. Clim. Chang. Strateg. Manag..

[61] Few R., Brown K., Tompkins E.L. (2007). Public participation and climate change adaptation: Avoiding the illusion of inclusion. Clim. Policy.

[62] van Aalst M.K., Cannon T., Burton I. (2008). Community level adaptation to climate change: The potential role of participatory community risk assessment. Glob. Environ. Chang..

[63] Hoff H., Warner K., Bouwer L.M. (2005). The Role of Financial Services in Climate Adaption in Developing Countries. Ierteljahrshefte Wirtsch..

[64] Sorgho R., Mank I., Kagoné M., Souares A., Danquah I., Sauerborn R. (2020). “We will always ask ourselves the question of how to feed the family”: Subsistence farmers’ perceptions on adaptation to climate change in Burkina Faso. Int. J. Environ. Res. Public Health.

[65] Seo S.N. (2010). Is an integrated farm more resilient against climate change? A micro-econometric analysis of portfolio diversification in African agriculture. Food Policy.

[66] Gabre-Madhin E., Haggblade S. (2003). Successes in African Agriculture: Results of an Expert Survey. World Dev..

[67] Sultan B., Labadi K., Guégan J.F., Janicot S. (2005). Climate Drives the Meningitis Epidemics Onset in West Africa. PLOS Med..

[68] Denton F. (2002). Climate change vulnerability, impacts, and adaptation: Why does gender matter?. Gend. Dev..

[69] Beg N., Morlot J.C., Davidson O., Afrane-Okesse Y., Tyani L., Denton F., Sokona Y., Thomas J.P., La Rovere E.L., Parikh J.K. (2002). Linkages between climate change and sustainable development. Clim. Policy.

[70] Fonta W.M., Ayuk E.T., van Huysen T. (2018). Africa and the Green Climate Fund: Current challenges and future opportunities. Clim. Policy.

[71] Nakhooda S., Caravani A., Bird N. (2011). Climate Finance in Sub-Saharan Africa.

[72] Smith J.B., Dickinson T., Donahue J.D., Burton I., Haites E., Klein R.J., Patwardhan A. (2011). Development and climate change adaptation funding: Coordination and integration. Clim. Policy.

[73] Ford J.D., Berrang-Ford L., Lesnikowski A., Barrera M., Heymann S.J. (2013). How to Track Adaptation to Climate Change: A Typology of Approaches for National-Level Application. Ecol. Soc..

[74] Austin S.E., Biesbroek R., Berrang-Ford L., Ford J.D., Parker S., Fleury M.D. (2016). Public Health Adaptation to Climate Change in OECD Countries. Int. J. Environ. Res. Public Health.

[75] Syrovátka M. (2009). Financing Adaptation to Climate Change in Developing Countries.

[76] Kim P.S. (2008). A Daunting Task in Asia. Public Manag. Rev..

[77] Wooden R. (2006). The Principles of Public Engagement: At the Nexus of Science, Public Policy Influence, and Citizen Education. Soc. Res. Int. Q..

[78] Finkelstein N.D. (2000). Introduction: Transparency in Public Policy. Transparency in Public Policy: Great Britain and the United States.

[79] Whitmarsh L., O’Neill S., Lorenzoni I. (2011). Engaging the Public with Climate Change: Communication and Behaviour Change.

[80] Lesnikowski A.C., Ford J.D., Berrang-Ford L., Barrera M., Heymann J. (2015). How are we adapting to climate change? A global assessment. Mitig. Adapt. Strateg. Glob. Chang..

[81] Lesnikowski A., Ford J., Biesbroek R., Berrang-Ford L., Heymann S.J. (2015). National-level progress on adaptation. Nat. Clim. Chang..

