# Addendum: Sorgho, R., et al. Climate Change Policies in 16 West African Countries: A Systematic Review of Adaptation with a Focus on Agriculture, Food Security, and Nutrition. *Int. J. Environ. Res. Public Health* 2020, *17*, 8897

**DOI:** 10.3390/ijerph18030945

**Published:** 2021-01-22

**Authors:** Raissa Sorgho, Carlos A. Montenegro Quiñonez, Valérie R. Louis, Volker Winkler, Peter Dambach, Rainer Sauerborn, Olaf Horstick

**Affiliations:** 1 Heidelberg Research to Practice Group, Heidelberg Institute of Global Health (HIGH), Heidelberg University Hospital, Heidelberg University, Im Neuenheimer Feld 365, 69120 Heidelberg, Germany; raissa.sorgho@uni-heidelberg.de (R.S.); carlos.montenegro@uni-heidelberg.de (C.A.M.Q.); Valerie.Louis@uni-heidelberg.de (V.R.L.); v.winkler@uni-heidelberg.de (V.W.); peter.dambach@uni-heidelberg.de (P.D.); 2 Working Group on Climate Change, Nutrition & Health, Heidelberg Institute of Global Health (HIGH), Heidelberg University Hospital, Heidelberg University, Im Neuenheimer Feld 324, 69120 Heidelberg, Germany; rainer.sauerborn@uni-heidelberg.de

The authors wish to make the following correction to this paper [1]:

In the original version of our article (Sorgho, R., et al. Climate Change Policies in 16 West African Countries: A Systematic Review of Adaptation with a Focus on Agriculture, Food Security, and Nutrition. 2020, 17(23), 8897), insufficient source of funding was given. The authors wish to change the information in the Funding section from:

**Funding**: This research was funded by German Academic Exchange Services, grant number 91727334.

to the correct version as follows:

**Funding**: This research was funded by the German Academic Exchange Services (DAAD), grant number 91727334, and by the Deutsche Forschungsgemeinschaft (DFG, German Research Foundation)—Projektnummer 427397520.

The authors would like to apologize for any inconvenience caused to the readers by these changes.
