# Supplementary material for: Climate Change Policies in 16 West African Countries: A Systematic Review of Adaptation with a Focus on Agriculture, Food Security, and Nutrition
Source: Int J Environ Res Public Health. 2020 Nov 30;17(23):8897. doi: 10.3390/ijerph17238897 (PMC7731384; doi:10.3390/ijerph17238897)
Supplement: Supplementary file 1 [file ijerph-17-08897-s001.zip › Sorgho_ijerph_SupplementaryMaterial/Sorgho_SR_SMTable3_2020.10.05.pdf]

	**Summary of Guideline Components**
Step 1<br><br>**Laying the ground work**	1) Initiating and launching the document writing procedure 2) Taking stock of currently available information on climate impact, adaptation and vulnerabilities while identifying the difficulties and gaps in the policy writing process 3) Addressing identified vulnerabilities, gaps and needs 4) Assessing development needs and climate vulnerabilities
Step 2<br><br>**Preparatory elements**	1) Analysing the current and future climate change scenarios of the country 2) Identifying at national, subnational and sector level climate vulnerabilities and adaptation options 3) Reviewing and evaluating adaptation options 4) Assembling the national plans for adaptation 5) Integrating policies into national and subnational policies, development and sectoral planning
Step 3<br><br>**Implementing Strategy**	1) Prioritizing climate change in the national planning 2) Developing long term implementation strategies for the adaptation plans 3) Increasing capacities for planning and implementation 4) Promoting climate adaptation plans in a coordinated and synergetic way in governmentally and multilaterally
Step 4<br><br>**Reporting, Monitoring and Review**	1) Creating a monitoring plan for the policy process 2) Reviewing and assessing the progress in filling the previously identified gaps 3) Updating the national agenda and national adaptation plans 4) Communicating the policy and continuously reporting on progress and effectiveness

Supplementary Material Table 3: The ssummary of the Steps Technical Guidelines for the NAP Process
