# Supplementary material for: Climate Change Policies in 16 West African Countries: A Systematic Review of Adaptation with a Focus on Agriculture, Food Security, and Nutrition
Source: Int J Environ Res Public Health. 2020 Nov 30;17(23):8897. doi: 10.3390/ijerph17238897 (PMC7731384; doi:10.3390/ijerph17238897)
Supplement: Supplementary file 1 [file ijerph-17-08897-s001.zip › Sorgho_ijerph_SupplementaryMaterial/Sorgho_SR_SMTable2_2020.10.05.pdf]

	Policy Document Name	Country
1	National Climate Change Adaptation Strategy	Cabo Verde
2	National Strategy for the Fight against Climate Change 2015-2020	Ivory Coast
3	The national strategy and action plan on climate change and variability 2003 (SN/PACVC)	Niger
4	National Climate Change Policy and Response Strategy	Nigeria
5	National Policy on Climate Change	Nigeria
6	Senegal's national policy to combat climate change	Senegal
7	National Climate Change Strategy And Action Plan (NCCSAP)	Sierra Leone
8	National Climate Change Policy (NCCP) 2015	Sierra Leone

Supplementary Material Table 3: The 8 policy documents for which the full text could not be located.
