# Supplementary material for: Climate Change Policies in 16 West African Countries: A Systematic Review of Adaptation with a Focus on Agriculture, Food Security, and Nutrition
Source: Int J Environ Res Public Health. 2020 Nov 30;17(23):8897. doi: 10.3390/ijerph17238897 (PMC7731384; doi:10.3390/ijerph17238897)
Supplement: Supplementary file 1 [file ijerph-17-08897-s001.zip › Sorgho_ijerph_SupplementaryMaterial/Sorgho_SR_SMTable1_2020.10.05.pdf]

Policy ID	Policy Document Title<br>&<br>Drafting Government Body	Country<br>&<br>Year	Vision Statement	CC Policy Integration Discussed?	CC Mitigation Discussed?
BN1	**Stratégie nationale de renforcement des ressources humaines, de l’apprentissage et du développement des compétences pour favoriser un développement vert, faible en émissions et résilient aux changements climatiques.**<br><br>*Ministère de l’Environnement Chargé de la Gestion des Changements Climatiques, du Reboisement et de la Protection des Ressources Naturelles et Forestières*	BN<br>2013	Le Bénin est, en 2025, un pays qui dispose de suffisamment de ressources humaines et d’institutions capables de contribuer, de manière efficace et efficiente, à la résolution des problèmes que posent les changements climatiques, afin de garantir un développement faible en émissions et résilient aux changements climatiques.	YES	YES
BN2	**Low Carbon and Climate Change Resilient Development Strategy 2016 – 2025**<br><br>*Ministère du Cadre de Vie et Du Develepement durable direction generale des changemement climatique*	BN<br>2016	La vision de la stratégie est ; « Le Bénin est, d’ici 2025, un pays dont le développement est résilient aux changements climatiques et à faible intensité de carbone ». Cette vision repose sur celle de BENIN ALAFIA 2025 et a pour objectif global de contribuer au développement durable du Bénin, par l’intégration des considérations climatiques dans les plans opérationnels sectoriels stratégiques du pays.	YES	YES
BF1	**The National Strategy for implementing the Climate Change Convention**<br><br>*Secrétariat Permanent du Conseil National pour la Gestion de l’Environnement (SP/CONAGESE)*	BF<br>2001	C'est dans le cadre de ce projet, que s'inscrit l'élaboration de la présente Stratégie Nationale initiale de Mise en Œuvre (SNMO) de la CCNUCC au Burkina Faso.Cette stratégie, issue de concertations nationales, doit permet-tre de mobiliser les audiences et les acteurs nationaux autour de straté- gies de développement intégrant les préoccupations liées aux change- ments climatiques *	YES	YES
IC1	**National Climate Change Program**<br><br>*Ministere de L’environement, de la salubrite Urbaine et du developement durable: Direction Générale de l’Environnement*	IC<br>2014	La vision consiste à la mise en place à l1horizon 2020 d1un cadre de développement socio- économique durable qui intègre les défis des changements climatiques dans tous les secteurs en Côte d1Ivoire et qui contribue à améliorer les conditions de vie des populations et leur résilience.	YES	YES

GB1	**National Climate Change Policy of The Gambia**<br><br>*Department of Water Resources Ministry of Environment, Climate Change, Water, Forestry and Wildlife*	GB 2016	Achieve a climate-resilient society, through systems and strategies that mainstream climate change, disaster risk reduction, gender and environmental management, for sustainable social, political and economic development.	YES	YES
GH1	**National Climate Change Policy**<br><br>*Ministry of Environment Science, Technology and Innovation (MESTI)*	GH 2013	Ensure a climate-resilient and climate-compatible economy while achieving sustainable development through equitable low-carbon economic growth for Ghana	YES	
GH2	**Guidebook for Mainstreaming of Climate Change and Disaster Risk Reduction for MMDAs**<br><br>*Environnemental Protection Agency*	GH 2010	This document is meant to guide the integration of climate change and disaster in all facets of national development policy and planning, from national, sector, regional and districts.	YES	YES
GNB1	**National Programme of Action of Adaptation to Climate Changes**<br><br>*Ministry of Natural Resources and Envronnment Government of Guinea-Bissau*	GNB 2006	Aware of the urgent need to prepare the country to tackle the threats and challenges that climate changes represent to mankind and life on earth, the government decided, as a major goal, to commit itself to the preparation of a National Program of Action of Adaptation to Climate Changes for Guinea-Bissau. It is a document axed on the country's short-medium term development objectives, which concur for the attainment of adaptation objectives, and thus raise their effectiveness. *	YES	YES
LB1	**Climate Change and Gender Action Plan for the Government of Liberia**<br><br>*Ministry of Gender and Development and Environmental Protection Agency*	LB 2012	To ensure that gender equality is mainstreamed into Liberia's climate change policies, programs and interventions so that both men and women have equal opportunities to implement and benefit from mitigation and adaptation initiatives in combating climate change and positively impact on the outcome of *"Liberia Rising 2030.* *	YES	YES
LB2	**National Policy and Response Strategy on Climate Change**<br><br>*Environmental Protection Agency of Liberia*	LB 2018	Liberia faces challenge with the impact of climate change coupled with many socio-economic problems like poverty, poor infrastructure, lack of information technology and access to finance, and weak institutions and resource competition, among others challenges. Several anthropogenic impacts have also worsened the climate change impact at the local level… In order to tackle the impact of climate change, which will effect a worsening situation on the development endeavor of the country, it has become vital that a climate	YES	YES

			change policy and response strategies, which address key sectorial and cross-sectorial issues, be put in place. *		
ML1	**Stratégie Nationales en matière de Changements Climatiques**<br><br>*Ministere de L'environnement de de L'assainissement*	ML 2011	Le projet « Stratégie Nationale Changements Climatiques Mali (SNCC Mali) » s`intègre dans cette démarche. Il a pour objectif de permettre au Mali d`avoir, de façon participative et consensuelle, une Stratégie Nationale CC. Cette stratégie serait déclinée en un Plan d`Action National Climat (PANC) intégrant les actions principales devant être menées par le Mali les années à venir en relation avec les CC *	YES	
ML2	**Cadre Stratégique pour une Economie Verte et Résiliente aux Changements Climatiques**<br><br>*Ministere de L'environnement de L'assainissement Agence de l'Environnement et du Développement durable (AEDD)*	ML 2001	La vision du Mali est de « disposer à horizon 2025, des modes d'organisation, de consommation et de production qui permettent à une population malienne sans cesse croissante de vivre durable- ment sur un espace territorial dont les ressources naturelles sont limitées et sous la contrainte des changements climatiques, faisant du pays un exemple en Afrique en matière de gestion des défis environnementaux et climatiques. »	YES	YES
ML3	**Politique Nationale sur les Changements Climatiques**<br><br>*Ministere de L'environnement de L'assainissement Agence de l'Environnement et du Développement durable (AEDD)*	ML 2011	La vision de la Politique Nationale sur les Changements Climatiques du Mali est de définir d'ici 2025 un cadre de développement socio-économique durable qui intègre les défis des changements climatiques dans tous les secteurs de son développement afin d'améliorer le bien être des populations. Elle se développera en s'orientant autour des cinq piliers opérationnels définis à Bali lors de la COP13 en 2007: la vision partagée, l'adaptation, l'atténuation, le transfert de technologies, et le financement, tout en associant de manière intégrée l'ensemble des programmations et l'ensemble des acteurs de la vie nationale.	YES	YES
ML4	**Stratégie Nationale Changements Climatiques : Plan d`Action National Climat**<br><br>*Ministere de L'environnement de L'assainissement Agence de l'Environnement et du Développement durable (AEDD)*	ML 2011	Le Plan Actions «rassemble l`ensemble des actions proposées selon les 8 axes stratégiques identifiés et dont la mise en œuvre est prévue pour 2012-2017 . IL s`agit du Plan d`Action National Climat Mali (PANC) 2012-2017 *	YES	
MR1	**Stratégie d'intégration de l'environnement et des changements climatiques dans le système éducatif mauritanien**	MR 2012	La présente réflexion a pour ambition de participer à cette action pour définir une stratégie qui devrait a terme contribuer, par l'éducation, au changement des mentalités et des comportements du mauritanien vis-à-vis des questions d'environnement et de développement durable. *		

	Ministere Delegue Aupres du Premier Ministre Charge de L'environement et du Developement Durable				
NG1	**Politique Nationale en matière de Changements Climatiques**<br><br>*Conseil National de l'Environnement pour un Développement Durable (*CNEDD*)*	NG 2012	Le Niger a décidé de se doter d'une politique nationale en matière de changements climatiques afin de coordonner les initiatives publiques dans ce domaine et qui servira de repère pour la prise en compte de cette dimension dans les politiques et stratégies de développement.La PNCC vise à contribuer à l'opérationnalisation de la vision des autorités nigériennes en matière de développement durable en se proposant, dans cette perspective, d'une part de s'affranchir des contraintes liées aux changements climatiques par la mise en œuvre généralisée et concertée des mesures d'adaptation et, d'autre part, d'introduire plus de responsabilité dans le processus de développement économique et social national par l'adoption des mesures d'atténuation susceptibles de réduire la tendance au réchauffement du climat.	YES	YES
NGA1	**National Adaptation Strategy and Plan of Action of Climate Change for Nigeria**<br><br>*Federal Ministry of Environment Special Climate Change Unit: Building Nigeria's Response to Climate Change (BNRCC)*	NGA 2011	This strategy envisions a Nigeria in which climate change adaptation is an integrated component of sustainable development, reducing the vulnerability and enhancing the resilience and adaptive capacity of all economic sectors and of all people -- particularly women, children, and resource-poor men – to the adverse impacts of climate change, while also capturing the opportunities that arise as a result of climate change.	YES	YES
NGA2	**National Policy of Climate Change**<br><br>*Federal Ministry of Environment*	NGA 2013	The vision of the National Climate Change Policy Response and Strategy (NCCPRS) is a climate change resilient Nigeria for rapid and sustainable socio-economic development. Its mission is to strengthen national initiatives to adapt to and mitigate climate change in a participatory manner involving all sectors of the Nigerian society, including the poor and other vulnerable groups (women, youth etc.) within the overall context of advancing sustainable socio-economic development in Nigeria.	YES	YES
TG1	**Plan National d'Adaption aux Changements Climatiques du Togo**<br><br>*Minister de L'environment et des Ressources Forestier*	TG 2016	À l'horizon 2030, le développement socioéconomique du Togo est durablement assuré et la résilience des populations vulnérables renforcée, grâce à la mise en œuvre des mesures d'adaptation aux changements climatiques. L'objectif global du PNACC est de contribuer à une croissance inclusive et durable au Togo à travers la réduction des vulnérabilités, le	YES	YES

			renforcement des capacités d'adaptation et l'accroissement de la résilience face aux changements climatiques.		

** Cases in which the policy document did not have a vision statement the goal or aim was recorded.

Supplementary Material Table 1: The first part of the policy extraction table
